# A Fully Integrated Low-Dropout Regulator with Improved Load Regulation and Transient Responses

**DOI:** 10.3390/mi13101668

**Published:** 2022-10-04

**Authors:** Chenkai Hu, Zhizhi Chen, Shenglan Ni, Qian Wang, Xi Li, Houpeng Chen, Zhitang Song

**Affiliations:** 1 The State Key Laboratory of Functional Materials for Informatics, Shanghai Institute of Microsystem and Information Technology, Chinese Academy of Sciences, Shanghai 200050, China; 2 University of Chinese Academy of Sciences, Beijing 100049, China

**Keywords:** fully integrated, load regulation, low-dropout regulator, fast-transient, system-on-chip (SoC), adjustable threshold push–pull stage, master–slave power transistors, low voltage

## Abstract

A fully integrated low-dropout (LDO) regulator with improved load regulation and transient responses in 40 nm technology is presented in this paper. Combining adjustable threshold push–pull stage (ATPS) and master–slave power transistors topology, the proposed LDO maintains a three-stage structure within the full load range. The proposed structure ensures the steady-state performance of LDO and achieves 0.017 mV/mA load regulation. The ATPS consumes little quiescent current at light load current condition, and the turn-on threshold of the ATPS can be adjusted by a current source. Once the value of current source is set, the turn-on threshold is also determined. A benefit of the proposed structure is that the LDO can be stable from 0 to 100 mA load current with a maximum 100 pF parasitic load capacitance and a 0.7 pF compensation capacitor. It also shows good figure of merit (FOM) without an extra transient enhanced circuit. For the maximum 100 mA load transient with 100 ns edge time, the undershoot and overshoot are less than 33 mV. The dropout voltage of the regulator is 200 mV with input voltage of 1.1 V. The total current consumption of the LDO was 24.6 μA at no load.

## 1. Introduction

The low-dropout linear regulator is a power converter that is widely used in power management, as it can provide low-ripple, low-noise and precision-regulated supply voltages for high-performance and noise-sensitive analog/mixed-signal blocks. The conventional PMOS LDO regulator, normally, needs a bulky off-chip capacitor in the range of several μF to achieve fast transient response and maintain stable [1,2]. For SoC application, removal of the off-chip capacitor can reduce the area of the printed circuit board (PCB) and the number of I/O pads on the chip, which is significantly beneficial in terms of integration. Therefore, in recent years, fully integrated LDO (or OCL-LDO) regulators have been widely studied and reported [3,4,5,6,7,8,9,10,11,12,13,14,15,16]. The output load capacitor $C_{L}$ mainly comes from parasitics of the power line, which is generally modeled from a few decades to 100 pF, and several orders lower than the off-chip capacitor. As a result, the major performance requirements of the fully integrated LDO will inevitably degrade aspects such as transient response and power-supply rejection (PSR). Therefore, the performance of a fully integrated LDO depends more on unity-gain bandwidth (UGB) and slew rate [15].

A series of technologies for improving the performance of fully integrated LDO are proposed. The push–pull stage is widely used to drive the power transistor in LDO regulators because the push–pull structure has greater driving ability [9,10]. LDO regulators making use of advanced compensation technology, which achieve more than 100 MHz UGB, have been proposed in [5,15]. However, it is worth noting that their load capacitor is limited below 5 pF, and their minimum load current is more than 120 μA. This is because if the load current is too low, the nondominant complex poles with a large Q factor cause a magnitude peaking near the unity gain frequency [16]. Thus, they are unattractive in low-power or large capacitive load applications. The flipped voltage follower (FVF) [12,13,14]-based LDO regulator is one of the most popular architectures due to its simplicity and its potential for fast transient response. In [14], an ultra-fast low-gain loop realized excellent transient response, and an additional loop is introduced to improve the DC accuracy. Nevertheless, its max load current is only 10 mA, and it consumes a large chip area to fabricate a 140 pF on-chip capacitor. Master–slave power transistors topology is popular in recent years, and it is used for ultra-low power design in [6,7], in which the LDOs transform between two-stage and three-stage cascaded topology at different load conditions. They can achieve ultra-low power consumption and good transient response. However, in order to maintain stable operation, the two-stage topology under light load comes at the cost of low accuracy. Especially in advanced processes, such as the 40 nm process, the small loop gain of LDO will lead to large dc error. In this paper, a LDO that combines master–slave power transistor topology and an adjustable threshold push–pull stage (ATPS) with improved transient response and load regulation is proposed.

## 2. Proposed LDO Regulator

### 2.1. Conventional Three-Stage LDO Regulators

**Figure 1:**
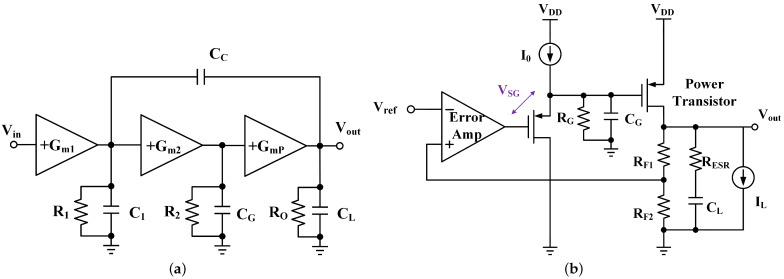
Conventional structure of LDO: (**a**) Three-stage LDO with Miller compensation; (**b**) buffer impedance attenuation based LDO regulator.

Conventional three-stage LDO regulators with single miller compensation can be modeled as Figure 1a. The dominant pole is located at the output of the first stage. Compared with the two-stage LDO regulator in [10], an additional stage $G_{m2}$ is added. Ignoring the presence of parasitic $C_{G}$, the LDO can be simplified as a second-order system with two poles. The second and the power stages together can be considered as a large $G_{m}$ stage with an effective $G_{m}$ of $G_{m2}$$R_{2}$$G_{mP}$, which is much higher than $G_{mP}$ alone, and the nondominant pole would be at $G_{m2}$$R_{2}$$G_{mP}$/$C_{L}$. However, this is an ideal assumption, because the decrease in the quiescent current leads to an increase in the impedance at each node and reduction in transconductance in each gain stage. The nondominant pole moves toward low frequency under both zero-load current, low quiescent current and large-load capacitor condition. Especially in less advanced processes, large $C_{G}$ makes the system third-order and the nondominant poles become complex [15] with large Q (=$R_{2}$$\sqrt{G_{m2}G_{mP}C_{gsP}/C_{L}}$). Complex poles locate at low frequency with large Q may lead to system instability [16].

As shown in Figure 1b, for a buffer impedance attenuation-based LDO regulator [1], the impedance ($R_{G}$) at the gate of the power transistor is attenuated by a buffer, such that the pole at the gate of the power transistor is pushed to high frequency. However, this kind of LDO regulator requires an additional $V_{SG}$ to ensure the operation. So, the LDO regulator struggles to fulfill the headroom budget in low-supply-voltage application [9]. Simultaneously, the gain of the buffer is approximately equal to one, so the buffer-based LDO regulator, in fact, is a two-stage LDO, and the loop gain is sacrificed.

### 2.2. Proposed ATPS

**Figure 2:**
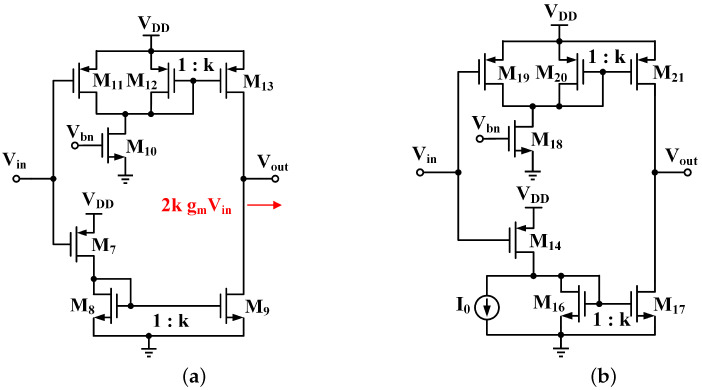
(**a**) Gm-boosting push–pull stage (**b**) proposed ATPS.

A gm-boosting push–pull stage is shown in Figure 2a. $M_{11}$ and $M_{12}$ have the same aspect ratio. $M_{12}$, $M_{13}$ and $M_{8}$, $M_{9}$ are two pairs of k-times current mirrors, and the effective transconductance is increased by 2k times. A push–pull output stage composed of $M_{13}$ and $M_{9}$ can charge and discharge the gate parasitic capacitance more effectively, since the bias current is increased by k times. To have a larger $g_{m}$ and driving ability, a larger proportionality factor k can be adopted, but at the expense of a quiescent current as the design trade off.

So, an adjustable threshold push–pull stage is proposed in this paper, as shown in Figure 2b. Compared with Figure 2a, ATPS has one more current source, $I_{0}$. Due to the existence of current source $I_{0}$, when the potential of $V_{in}$ is relatively high, the current of $M_{14}$ and $M_{19}$ is small. The drain of $M_{14}$ is pulled to the ground; thus, $M_{17}$ has no current, and the drain of $M_{21}$ is pulled to power $V_{DD}$. At this time, the ATPS is turned off and only $M_{18}$ and $I_{0}$ consume very little quiescent current. The turn-on threshold can be adjusted by the value of $I_{0}$. Once the fixed bias $I_{0}$ is set, the turn-on threshold is also determined. When the ATPS turned on, it works like the $g_{m}$ boosting push–pull stage. With a large k, $g_{m}$ and driving ability significantly improved, without significantly increasing the quiescent current under light load.

### 2.3. Circuit Implementation

**Figure 3:**
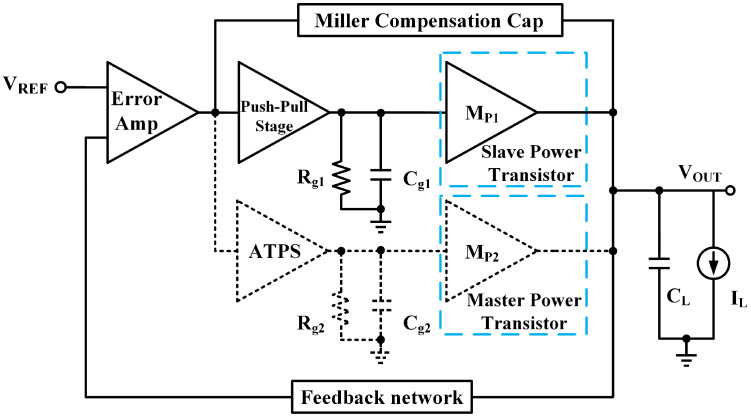
Simplified block diagram of the proposed topology.

**Figure 4:**
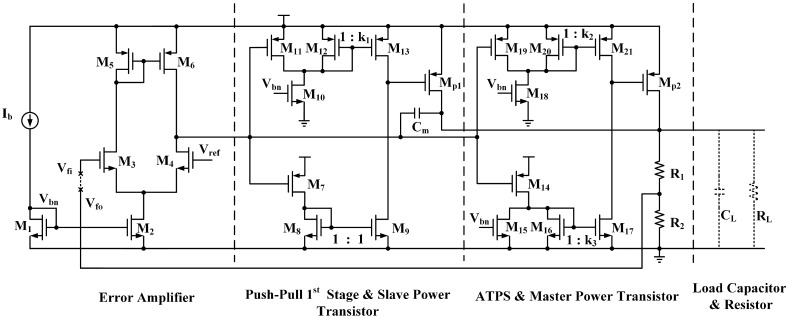
Schematic of the proposed LDO regulator.

A simplified structure block diagram is shown in Figure 3. The corresponding schematic of the regulator is depicted in Figure 4. The gm boosting push–pull stage and ATPS correspond to $M_{7}$–$M_{13}$ and $M_{14}$–$M_{21}$, respectively. The feedback factor, β = $R_{1}$*⁄*($R_{1}$ + $R_{2}$), is 5/9 in this design and the reference voltage $V_{ref}$ is 500 mV. $M_{2}$–$M_{6}$ form the differential input stage. The aspect ratio of $M_{p2}$ is 60 times that of $M_{p1}$. In this design, the turn-on threshold of ATPS is designed to be $I_{LOAD}$ = 500 μA by setting the current of $M_{15}$ to 2.5 μA.

When load current is less than about 500 μA, the ATPS and $M_{p2}$, dotted line in the Figure 3, is off. When load current is more than about 500 μA, the ATPS turns on and two power transistors work together to provide load current. Compared with [1,6,7], the structure proposed in this letter maintains a three-stage structure within the full load range rather than two-stage or three-stage cascaded topology at different load conditions. The proposed structure ensures the steady-state performance of LDO, such as load regulation. Compared with conventional LDO at light load condition, since the master power transistor is turned off, the gate parasitic capacitance of the power transistor with large aspect ratio can be considered “reduced”. So, the Q is reduced at light load condition. The parasitic capacitance is related to the nondominant poles, which also means the nondominant pole in this structure is moved to a higher frequency, benefiting from frequency compensation. When the load current increases, the potential at the output of the error amplifier decreases and the ATPS turns on. Then, the current in $M_{17}$ and $M_{21}$ naturally increases. Therefore, they can drive the power transistor more effectively.

**Figure 5:**
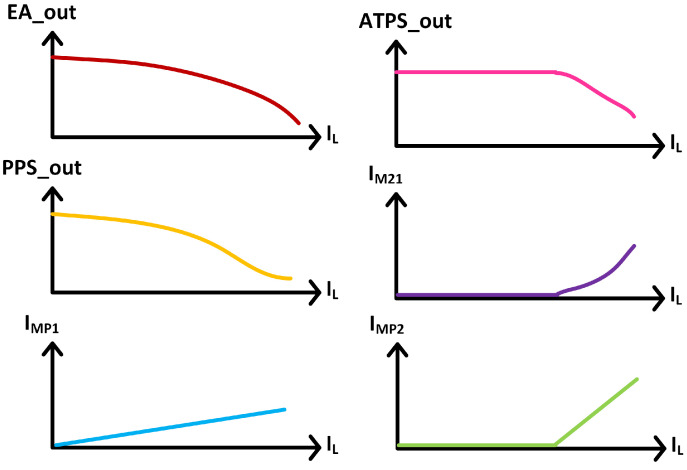
Detailed overall operating waveform of the proposed LDO.

The detailed overall operating waveform of the proposed LDO is shown in Figure 5. EA_out, PPS_out and ATPS_out are the output voltage of error amp, push–pull stage and ATPS, respectively, in Figure 3; $I_{MP1}$ and $I_{MP2}$ are the current of $M_{P1}$ and $M_{P2}$, respectively, in Figure 3; $I_{M21}$ is the current of $M_{21}$ in Figure 4. When the LDO is under light load condition, the ATPS is off. So ATPS_out, $I_{MP2}$ and $I_{M21}$ remain unchanged, and only $M_{P1}$ provides current for the load. When load current is more than 500 μA, ATPS is on and $M_{P1}$ and $M_{P2}$ provides current for the load together. Meanwhile, $I_{M21}$ increases as the load current increases, which improves transient response under heavy load.

### 2.4. Stability Analysis

**Figure 6:**
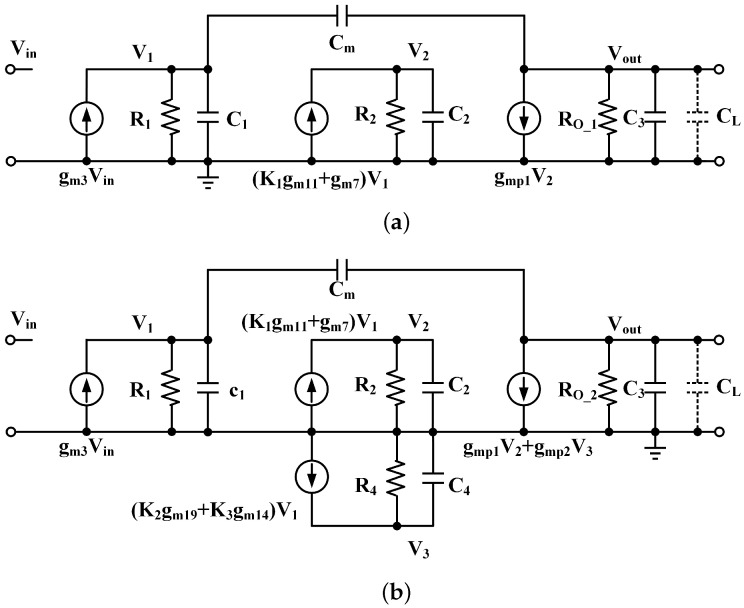
Small-signal model of the proposed LDO regulator: (**a**) ATPS off (**b**) ATPS on.

The stability of the LDO regulator is realized by single miller compensation. Due to the structural transformation, the stability of the proposed fully integrated LDO regulator will be discussed on the basis of ATPS on and off structure, as shown in Figure 6. The transfer function is derived using the following assumptions: (a) the gains in the first stage, push–pull stage and ATPS are much larger than one, (b) $g_{mi}$ is defined as the transconductance of the respective device, $C_{i}$ and $R_{i}$ denote the respective lumped output parasitic capacitance and output resistance of each node, (c) the capacitances $C_{L}\gg C_{m}$, $C_{4}\gg C_{1}$, $C_{2}$, (d) $g_{mp2}\gg g_{mp1}$.

Case I ($I_{LOAD}$ < 500 μA): When $I_{LOAD}$ < 500 μA the ATPS is off, the gate’s potential of the $M_{p2}$ is pulled to power $V_{DD}$. Thus, ATPS and $M_{p2}$ can be ignored in the analysis of Case I. Figure 6a shows the small-signal model, which is similar to Figure 1a, except for the parasitic capacitor at the gate of the power transistor. The effective output resistance for Case I is $R_{o_{-}1}$ = $r_{oMp1}$//$R_{FB}$//$R_{LOAD}$, where $r_{oMp1}$, $R_{FB}$ and $R_{LOAD}$ are the output resistance of the slave–power transistor, feedback network resistance and load resistance, respectively. The derived transfer function is shown as Equation (1).

(1) $$A_{V\left(I_{LOAD}<500\mu A\right)}=\frac{-\beta g_{m3}G_{1}g_{mP1}R_{o_{-}1}R_{2}R_{1}\left(1-\frac{C_{m}}{G_{1}R_{2}g_{mP1}}S-\frac{C_{m}C_{2}}{G_{1}g_{mP1}}S^{2}\right)}{\left(1+R_{1}C_{m}G_{1}R_{2}g_{mP1}R_{O_{-}1}S\right)\left(1+\frac{C_{L}}{G_{1}R_{2}g_{mP1}}S+\frac{C_{L}C_{2}}{G_{1}g_{mP1}}S^{2}\right)}$$

where $G_{1}=K_{1}g_{m11}+g_{m7}$. Because $C_{2}\ll C_{L}$, the three poles are separated real poles. The low-frequency gain $A_{V0}$ and dominant pole $p_{-3\mathrm{dB}}$ are given as

(2) $$A_{V0}=-g_{m3}G_{1}g_{mP1}R_{o_{-}1}R_{2}R_{1}$$

(3) $$p_{-3\mathrm{dB}}=-\frac{1}{R_{1}C_{m}G_{1}R_{2}g_{mP1}R_{o_{-}1}}$$

The gain-bandwidth product is given by $GBW=g_{m3}/C_{m}$. The nondominant poles can be given as $p_{2}=-G_{1}R_{2}g_{mP1}/C_{L}$, $p_{3}=-1/\left(R_{2}C_{2}\right)$. Since the zeros are located at a higher frequency, they are neglected. The worst PM occurs when the load current is zero and the load capacitance is 100 pF, because $p_{2}$ is inversely proportional to $C_{L}$ and proportional to $g_{mp1}$. Additionally, $g_{mp1}$ is proportional to the square root of the load current. Thus, the PM is enhanced when the load current increases. The $p_{3}$ is located at higher frequency and has little impact on PM. The PM can be derived as

(4) $$\mathrm{PM}=180^{\circ }-\tan^{-1}\left(\frac{GBW}{p_{-3\mathrm{dB}}}\right)-\tan^{-1}\left(\frac{GBW}{p_{2}}\right)$$

From Equations (3) and (4), we see that as $C_{2}$ decreases and $p_{3}$ is pushed to higher frequency, the minimum $C_{m}$ required is reduced.

Case II ($I_{LOAD}$ ≥ 500 μA): When $I_{LOAD}$ ≥ 500 μA the ATPS is on, both ATPS and $M_{p2}$ should be considered in the stability analysis. Figure 6b shows the small-signal model. $R_{o_{-}2}$ = $r_{oMp1}$//$r_{oMp2}$//$R_{FB}$//$R_{LOAD}$ is the effective output resistance for Case II, where $r_{oMp2}$ is the resistance of the master power transistor. The transconductance $g_{mp2}$ is much larger than $g_{mp1}$. The derived transfer function is shown as Equation (5), $G_{2}=K_{2}g_{m19}+K_{3}g_{m14}$.

(5) $$A_{V\left(I_{LOAD}>500\mu A\right)}=\frac{-\beta g_{m3}R_{1}GR_{o_{-}2}\left[1+\left(\frac{G_{1}R_{2}g_{mP1}C_{4}R_{4}}{G}\right)S-\left(\frac{C_{m}C_{4}R_{4}}{G}\right)S^{2}-\left(\frac{C_{m}C_{2}R_{2}C_{4}R_{4}}{G}\right)S^{3}\right]}{\left(1+R_{1}C_{m}GR_{o_{-}2}S\right)\left(1+\frac{G_{1}R_{2}g_{mP1}C_{4}}{G_{2}g_{mP2}}S+\frac{R_{2}C_{4}C_{2}}{G_{2}g_{mP2}R_{o_{-}2}}S^{2}\right)\left(1+R_{o_{-}2}C_{L}S\right)}$$

Because $C_{2}\ll C_{L}$, the three poles are separated real poles. The low-frequency gain $A_{V0}$ and dominant pole $p_{-3\mathrm{dB}}$ are given as

(6) $$A_{V0}=-g_{m3}R_{1}GR_{o_{-}2}$$

(7) $$p_{-3\mathrm{dB}}=-\frac{1}{R_{1}C_{m}GR_{o_{-}2}}$$

The nondominant complex poles can be approximately derived as

(8) $$\left|p_{2,3}\right|=\sqrt{\frac{G_{2}g_{mP2}R_{O_{-}2}}{R_{2}C_{4}C_{2}}}$$

From Equation (8), $\left|p_{2,3}\right|$ relies on $\sqrt{g_{mP2}R_{o_{-}2}}$ and locates at high frequency. A higher frequency pole locates at $p_{4}=-\frac{1}{R_{o_{-}2}C_{L}}$. Since zeros are located at a higher frequency, they are neglected. Similar to [6,9,16], the worst PM occurs when $I_{L}OAD$ is minimum and $C_{L}$ is maximum, so the LDO can be stable as long as $C_{L}$ is less than 100 pF.

## 3. Simulation Results and Discussion

### 3.1. Open-Loop Frequency Response

**Figure 7:**
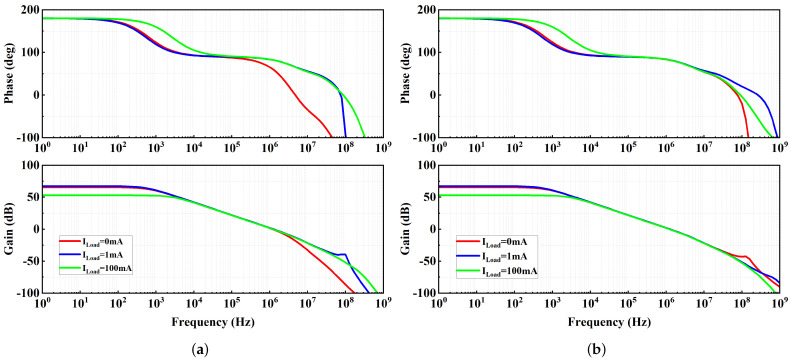
Simulated open-loop frequency response at different $I_{LOAD}$: (**a**) $C_{L}$ = 100 pF; (**b**) $C_{L}$ = 0 pF.

**Figure 8:**
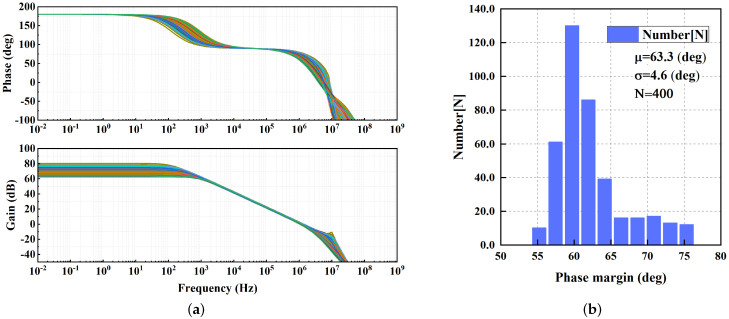
Monte Carlo simulation (400 runs) for mismatch and process variations: (**a**) Simulated open-loop frequency response. $I_{L}$ = 0 mA, $C_{L}$ = 100 pF; (**b**) Phase margin.

The simulated open-loop frequency responses of the proposed LDO regulator at different Load conditions are shown in Figure 7. The regulator achieves a minimum phase margin of 60° with a 100 pF load capacitor. As previously analyzed, PM increases with the increase in the load current. To verify the stability when the load capacitance is zero, open-loop frequency responses are simulated and shown in Figure 7b. A better PM is achieved, because nondominant poles are shifted to higher frequencies. The result of the 400-run Monte Carlo analysis for mismatch and process variations is shown in Figure 8. The μ and σ of phase margin are 63.3° and 4.6°, respectively.

### 3.2. Load Transient Response, Load Regulation, Line Transient Response

**Figure 9:**
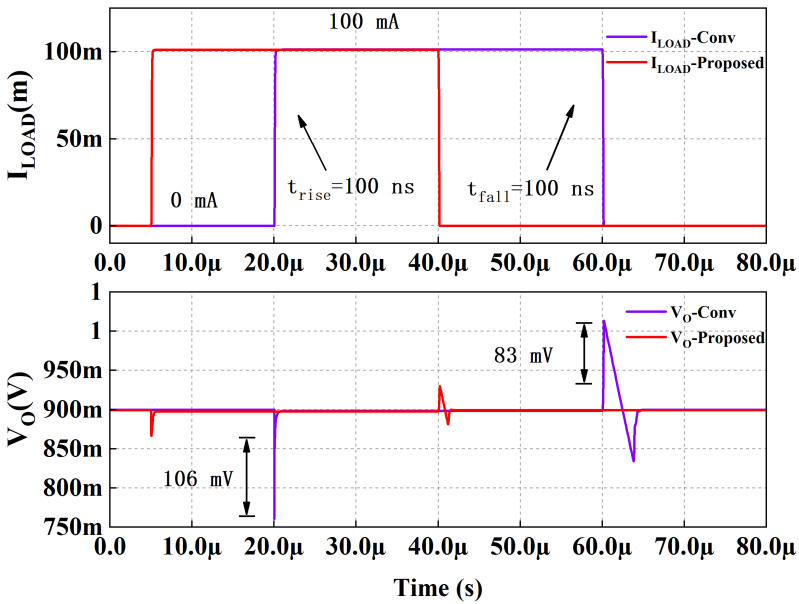
Simulated load transient response with 0–100 mA load current step. $V_{DD}$ = 1.1 V, $V_{OUT}$ = 0.9 V, $C_{L}$ = 100 pF.

**Figure 10:**
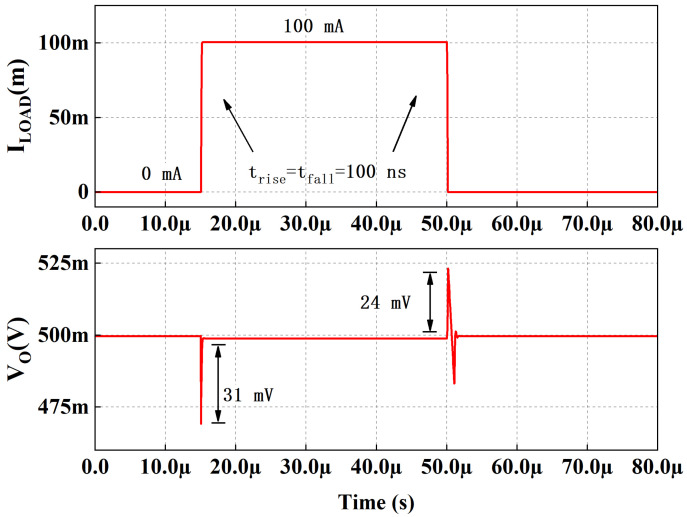
Simulated load transient response with 0–100 mA load current step. $V_{DD}$ = 1.1 V, $V_{OUT}$ = 0.5 V, $C_{L}$ = 100 pF.

Figure 9 illustrates the load transient response with a full load current step from 0 A to 100 mA at the edge time of 100 ns of proposed LDO and conventional LDO. The conventional LDO is a three-stage LDO with a $g_{m}$-boosting push–pull stage as the second stage. The quiescent current of proposed LDO and conventional LDO are the same at no load. The undershoot and overshoot of the proposed LDO are 32 mV and 33 mV, respectively, and are better than conventional LDO. The reference voltage, $V_{ref}$, is 0.5 V, so the minimum output voltage is 0.5 V when feedback is unit gain negative feedback. Figure 10 shows the load transient response with 0–100 mA load current step at the edge time of 100 ns of the proposed LDO when $V_{DD}$ = 1.1 V, $V_{OUT}$ = 0.5 V, $C_{L}$ = 100 pF. The undershoot and overshoot are 31 mV and 24 mV, respectively.

**Figure 11:**
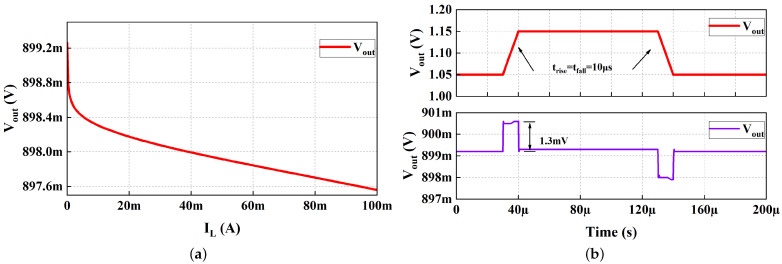
(**a**) Simulated load regulation of the proposed fully integrated LDO with $V_{I}N$ = 1.1 V and $V_{OUT}$ = 0.9 V; (**b**) line transient response with $V_{DD}$ step between 1.05 and 1.15 V.

Figure 11a shows the load regulation of the proposed work, which is 0.017 mV/mA. The line transient response is simulated at no-load current, with the supply voltage switching between 1.05 and 1.15 V at an edge time of 10 µs. Figure 11b depicts the voltage spike as 1.3 mV in the line transient simulation.

### 3.3. ATPS

**Figure 12:**
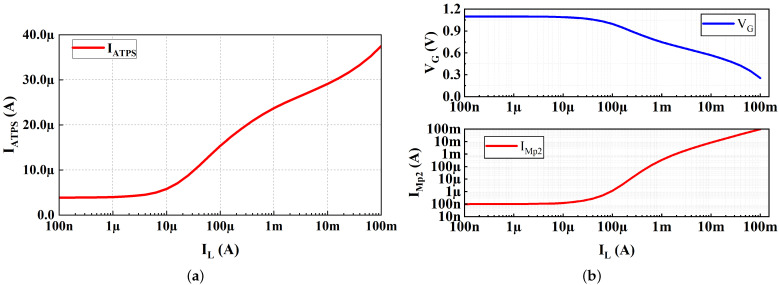
(**a**) Simulated quiescent current of ATPS versus $I_{L}$; (**b**) gate potential ($V_{G}$) of $M_{p2}$ and current of $M_{p2}$($I_{Mp2}$) versus $I_{L}$.

The quiescent current of ATPS is the current of $M_{14}$, $M_{18}$ and $M_{21}$. As shown in Figure 12a, in the off state, the quiescent current of ATPS is 3.8 μA. With the increase in the load current, the quiescent current of ATPS will increase to 37 μA. As previously analyzed, the dynamic bias strategy of ATPS not only improves the efficiency under light load, but also improves the transient response under heavy load.

As shown in Figure 12b, with the increase in the load current, $V_{G}$ remains unchanged and then decreases. With the increase in load current, $I_{Mp2}$ remains unchanged and then decreases. $V_{G}$ is the gate voltage of $M_{p2}$ and also the output of ATPS; $I_{Mp2}$ is the current of $M_{p2}$. The simulation results verify the previous analysis: the gate of the power transistor $M_{p2}$ is pulled to $V_{DD}$ by ATPS, and the $M_{p2}$ turns off under light load.

### 3.4. Power-Supply Rejection

The PSR of a LDO can be given as [17]

(9) $$\mathrm{PSR}=\frac{v_{out}\left(s\right)}{v_{in}\left(s\right)}=\frac{v_{out}\left(s\right)}{v_{in}\left(s\right)}=\frac{\frac{R_{L}}{R_{L}+r_{ds}}}{\left(1+\frac{s}{\omega_{o}}\right)\left(1+LG\left(s\right)\right)}$$

where $\omega_{o}$ is the pole at the output of the LDO, LG(s) is the loop gain and $R_{L}$ and $r_{ds}$ denote the load resistance and the output impedance of $M_{P}$, respectively. If the dominant pole is inside the loop and the output is the nondominant pole, loop gain rolls off at the −20 dB/decade slope, causing the PSR to degrade at the same rate from $\omega_{dominant}$. This degradation continues until the loop-gain unity-gain frequency, $\omega_{ugb}$, after which PSR remains flat because the ripple is only reduced by the resistive divider formed between $R_{L}$ and $r_{ds}$ [17].

**Figure 13:**
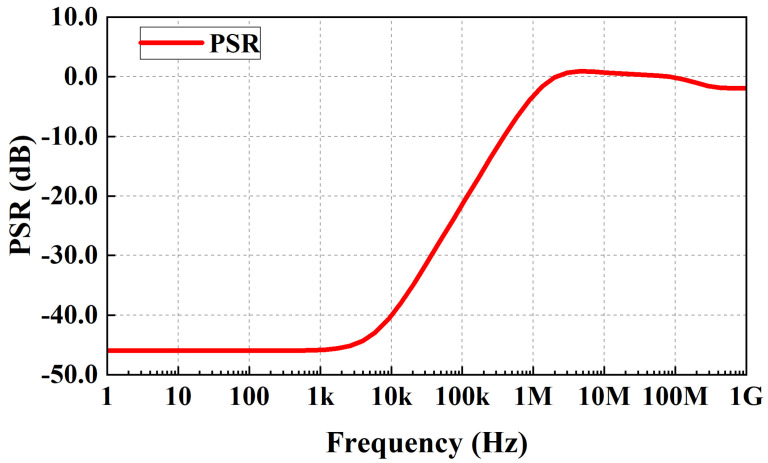
Simulated PSR performance of the proposed LDO at 100-mA load current, 0-pF $C_{L}$ and 200-mV dropout.

Simulated PSR performance of the proposed LDO at 100 mA load current, 0-pF $C_{L}$ and 200 mV dropout is shown in Figure 13. The PSR of the proposed LDO is −46 dB at 1 KHz and −2.5 dB at 1.1 MHz. The PSR degrades at −20 dB/decade from $\omega_{dominant}$ (about 5 kHz) and remains flat after $\omega_{ugb}$ (about 1.1 MHz), which corresponds to the analysis in [17] and the simulated open-loop frequency response in Figure 7b. In Figure 7b, the dominant pole and the unity-gain bandwith is located at about 5 kHz and 1.1 MHz, respectively.

### 3.5. Performance Comparison

For different processes, the minimum channel length (L) will affect the parasitic capacitance of the power transistor. If a process has a shorter minimum L, the FOM could be smaller owing to the smaller parasitic capacitance of the transistor. For fair comparison, the figure-of-merit (FOM) equation, as given below, which was originally proposed in [11], considering minimum L is adopted to compare the transient response.

(10) $$\mathrm{FOM}=T_{edge}\cdot \Delta V_{OUT}\cdot \left(I_{Q}+I_{LOAD(min)}\right)/\left(\Delta I_{LOAD}\cdot L^{2}\right)$$

The performance comparison of the proposed LDO with several state-of-the-art fully integrated LDOs is shown in Table 1. The proposed LDO has achieved quite comparable load regulation and FOM.

## 4. Conclusions

A transient-enhanced, fully integrated LDO regulator is presented in this paper. Through the combination of ATPS and master–slave power transistor topology, the LDO regulator can achieve good transient response, without significantly increasing quiescent current at light load. In the full load range, the LDO always maintains a three-stage structure, which ensures the loop gain and accuracy and achieves good load regulation. The proposed fully integrated LDO regulator achieves stability from 0 to 100 mA without the minimum load current limit. The miller compensation capacitor for stability can be reduced, as well.

## Figures and Tables

**Table 1:** Performance comparison of the proposed LDO with several state-of-the-art fully integrated LDO regulators.

Parameters	This Work	[3]	[9]	[8]	[4]
Year	2022	2020	2022	2014	2017
Technology(nm)	40	65	65	350	40
$I_{LOAD(max)}$(mA)	100	100	50	100	200
$I_{LOAD(min)}$(mA)	0	0	0	0.01	0
$V_{IN}$(V)	1.1	0.95–1.2	0.75–1.2	2.7–3.3	1.1
$V_{OUT}$(V)	0.9	0.8	0.5	2.5	1
$C_{on-chip}$(pF)	0.7	6	2	14	4
$C_{L}$(pF)	0–100	0–100	0–100	0–100	0–100
PSR(dB@kHz)	−46@1	−33@10	−46@1	−41@10	−66@100
$I_{Q}$(μA)	24.6–65	14	16.2	66	275
Δ$V_{OUT}$(mV)	33	230	103	255	124
Edge Time(ns)	100	220	100	400	100
Load Regulation(mV/mA)	0.017	0.09	0.48	0.06	0.019
FOM(ns· V/μm^2^) *	0.507	1.67	0.79	0.632	10.65

[*] $\mathrm{FOM}=T_{edge}\cdot \Delta V_{OUT}\cdot \left(I_{Q}+I_{LOAD(min)}\right)/\left(\Delta I_{LOAD}\cdot L^{2}\right)$ proposed in [11].

## Data Availability

Not applicable.
